# Using Deep Learning in a Monocentric Study to Characterize Maternal Immune Environment for Predicting Pregnancy Outcomes in the Recurrent Reproductive Failure Patients

**DOI:** 10.3389/fimmu.2021.642167

**Published:** 2021-04-01

**Authors:** Chunyu Huang, Zheng Xiang, Yongnu Zhang, Dao Shen Tan, Chun Kit Yip, Zhiqiang Liu, Yuye Li, Shuyi Yu, Lianghui Diao, Lap Yan Wong, Wai Lim Ling, Yong Zeng, Wenwei Tu

**Affiliations:** ^1^Department of Pediatric and Adolescent Medicine, Li Ka Shing Faculty of Medicine, The University of Hong Kong, Hong Kong, China; ^2^Shenzhen Key Laboratory of Reproductive Immunology for Peri-Implantation, Fertility Center, Shenzhen Zhongshan Urology Hospital, Shenzhen, China; ^3^ALOM Intelligence Limited, Hong Kong, China

**Keywords:** artificial intelligence, recurrent reproductive failure, reproductive immunology, sparse coding, assisted reproductive technology

## Abstract

Recurrent reproductive failure (RRF), such as recurrent pregnancy loss and repeated implantation failure, is characterized by complex etiologies and particularly associated with diverse maternal factors. It is currently believed that RRF is closely associated with the maternal environment, which is, in turn, affected by complex immune factors. Without the use of automated tools, it is often difficult to assess the interaction and synergistic effects of the various immune factors on the pregnancy outcome. As a result, the application of Artificial Intelligence (A.I.) has been explored in the field of assisted reproductive technology (ART). In this study, we reviewed studies on the use of A.I. to develop prediction models for pregnancy outcomes of patients who underwent ART treatment. A limited amount of models based on genetic markers or common indices have been established for prediction of pregnancy outcome of patients with RRF. In this study, we applied A.I. to analyze the medical information of patients with RRF, including immune indicators. The entire clinical samples set (561 samples) was divided into two sets: 90% of the set was used for training and 10% for testing. Different data panels were established to predict pregnancy outcomes at four different gestational nodes, including biochemical pregnancy, clinical pregnancy, ongoing pregnancy, and live birth, respectively. The prediction models of pregnancy outcomes were established using sparse coding, based on six data panels: basic patient characteristics, hormone levels, autoantibodies, peripheral immunology, endometrial immunology, and embryo parameters. The six data panels covered 64 variables. In terms of biochemical pregnancy prediction, the area under curve (AUC) using the endometrial immunology panel was the largest (AUC = 0.766, accuracy: 73.0%). The AUC using the autoantibodies panel was the largest in predicting clinical pregnancy (AUC = 0.688, accuracy: 78.4%), ongoing pregnancy (AUC = 0.802, accuracy: 75.0%), and live birth (AUC = 0.909, accuracy: 89.7%). Combining the data panels did not significantly enhance the effect on prediction of all the four pregnancy outcomes. These results give us a new insight on reproductive immunology and establish the basis for assisting clinicians to plan more precise and personalized diagnosis and treatment for patients with RRF.

## Introduction

Pregnancy is a complex biological process that poses a great challenge to the maternal immune system. The unique immunology of the maternal–fetal interface was recognized, since the “fetal allograft” concept was first described by Sir Peter Brian Medawar in the early 1950s (1). Correct and precise cross-talk between fetus and mother is an important basis for the apposition, adhesion, implantation, and growth of the embryo in the uterus (2). The abnormal frequencies and functions of maternal immune cells are associated with reproductive failure, especially in cases of recurrent reproductive failure (RRF), such as recurrent pregnancy loss (RPL) and repeated implantation failure (RIF) (3).

In the conventional medical procedure, the patients with RRF are assessed and given a score, based on biomarkers that have been demonstrated to be of relevance to the disease. The treatment for the patients is based on the classification or scores (4, 5). However, the etiologies of RRF are highly heterogeneous and the complex underlying interactions between the biomarkers make the creation of a personalized treatment strategy based on all known parameters impossible for the clinicians. Therefore, the design of a model that can accurately predict the outcome of treatment methods would be highly beneficial to the clinicians, enabling the choice of lower-risk treatments, thus alleviating the financial burden of the treatment cost and reducing treatment time.

In the field of assisted reproductive technology (ART), predictive models have been applied as decision aids to embryo, egg, sperm selection, and pregnancy outcome prediction, and for the intrinsic evaluation of various factors related to clinical outcomes (6, 7). Presently, the validity of the applied models has been demonstrated by analyzing the correlation between factors and the treatment outcome or etiology (8). However, the varying degrees of accuracy and limitations of the applied models have inhibited their use in the routine implementation of *in vitro* fertilization (IVF) procedures (6). To address this problem, more complex Artificial Intelligence (A.I.) systems, such as artificial neural networks (ANNs), have been introduced in ART fields (9, 10). A.I. systems are advantageous due to their significant information processing properties in terms of non-linearity, high levels of parallelism, noise and fault tolerance, as well as learning, generalization, and adaptive capabilities (11). Nevertheless, few studies that focus on the pregnancy outcome prediction in patients with RRF exist.

Sparse coding is a common machine learning technique used to extract features from raw data. The core of sparse coding involves establishing a sparse representation of the raw data to form a linear combination of basic elements called “atoms,” which collectively form a library known as “dictionary.” The advantages of using sparse coding include: (1) training a learning model by adopting a relatively lower number of features from raw data, which, in turn, lowers the computational cost during model training; (2) increased interpretability of the learning results as critical features that can be identified efficiently from the dictionary (12). It has been demonstrated that sparse coding can be applied in genome-wide association studies, neuroimaging, and oncology for object detection and classification tasks (13–16). Sparse coding techniques have not been comprehensively studied in the area of reproductive medicine, and its application in immunological profile analysis of patients with RRF remains to be explored.

The remainder of this article is organized as follows. A literature review in the field of A.I. and reproductive medicine is presented in the next section, followed by the methodology and demonstration of sparse coding application to analyze multidimensional clinical data of patients with RRF and predict their pregnancy outcomes. The results on the performance of the model are then introduced and finally, the concluding remarks are presented in the discussion.

### Overview of A.I. Application in Reproductive Failure

#### Artificial Intelligence in Predicting Pregnancy Outcomes of Patients With Infertility

Machine learning is a subset of A.I. that enables computer algorithms to model the relationship between a set of observable data (input data) and another set of variables (output data) (17). It provides the ability to interpret and understand data and to develop predictive models based on experience. Machine learning methods include ANN, Support Vector Machines (SVM), C4.5, Classification and Regression Tree (CART), Random Forest (RF), K-Nearest-Neighbor (KNN), and so on. ANNs and SVMs are widely used in biomedical problems analysis. Machine learning methods can provide more options and richer task information for problem solving. At the same time, machine learning methods are gaining popularity in clinical decision-making (18–20).

The concept of a neural network is derived from the structure and function of biological neural networks. In particular, ANNs propose a system with stacked layers of interconnected processors, or nodes, that can form increasingly complex features in each successive layer (21). Raw information is supplied into the input layer and passes through the implicit layer by a weighted connection system. Finally, the output values of the transformed features are generated in the output layer, to predict the outcome. In a clinical setting, the input layer can represent medical data, the output layer can represent prognostic subclasses and multiple implicit layers can represent feature detectors, used to capture higher-order correlations. The SVM algorithm classifies the input data by calculating support vectors that construct hyperplanes in a higher-dimensional space, where the features are separable. C4.5, CART, and RF are three decision trees with non-parametric characteristics that map characteristics to outcomes using a partitioning procedure that recursively divides the source set of each node or branch point into unrelated subsets based on the value of a particular characteristic. KNN is an instance-based learning method that assigns classes to the data, based on nearest-neighbor decision rules.

On the basis of big data training iterations, dimensionality reduction can be applied to a large number of influential factors by using various machine learning methods for modeling and prediction. Simultaneously, relevant attributes with high influence can be extracted, and a prediction model with relatively high accuracy can be obtained. Hassan et al. (21) evaluated the predictive ability of five different machine learning models, namely, Multilayer Perceptron (MLP), SVM, C4.5, CART, and RF, on the success rate of IVF pregnancies. A feature selection algorithm for climbing features (attributes), combined with automatic classification using a machine learning technique, was used to reduce the number of most influential attributes to 19 for MLP, 16 for RF, 17 for SVMs, 12 for C4.5, and 8 for CART, in order to analyze and predict IVF pregnancies in a more accurate manner. The most influential factors were summarized as: age, fertility factor index, basal sinus follicle count, number of mature eggs, sperm collection method, gametes, *in vitro* fertilization rate, 14-day follicle count, and embryo transfer date. Vogiatzi et al. (8) used the ANN approach to validate the efficiency of an ANN based on correlated parameters of live birth as a comprehensive tool for predicting clinical outcomes in patients undergoing ART. The ANN was constructed using 12 statistically significant parameters from the initial integration with a cumulative sensitivity of 76.7% and a specificity of 73.4%. The standard deviation of the performance metrics evaluated between the training and the testing sets was low in the validation process, pointing to the stability of the constructed ANN. The constructed ANN, based on statistically significant live birth outcome variables, is a stable and efficient system with increased performance metrics. The validation of the system led to the recognition of its clinical value as a medical decision aid and provided a reliable method for the routine practice of IVF units in a user-friendly environment. Elson et al. (22) developed a decision tree based on a combination of clinical, morphological, and biochemical parameters predicting successful pregnancy outcomes that assisted the expected management of women with tubal ectopic pregnancies. Significant differences were detected in maternal age, initial serum β-hCG, and progesterone among pregnant women who required surgery or recovered on their own. Analysis utilizing a decision tree can be used as an estimation guide for the probability of successful prediction individually.

Machine learning methods have, in general, high prediction accuracy; however, the final model prediction accuracy can vary. Commonly used classifiers include SVMs, recursive partitioning, RF, adaptive augmentation, and KNN. Hafiz et al. (23) used data mining techniques to predict the implantation outcome of IVF and intracytoplasmic sperm injection (ICSI), which were found to be superior to other comparable methods using RF and recursive partitioning, with the corresponding area under the ROC curve (AUC) values reaching 84.23 and 82.05%, respectively. Ghaeini et al. (24) proposed an ICSI outcome prediction model based on decision trees and SVMs. The input variables of the model included parameters such as the medical history of the couple, hormone testing, and cause of infertility. The output variable was the occurrence of a clinical pregnancy. The accuracies of the decision tree method and SVMs were 70.3 and 75.7%, respectively. The performance of the SVM method was superior to the performance of the decision tree method.

#### Artificial Intelligence in Predicting Pregnancy Outcomes of Patients With RRF

Currently, research on predictive models for pregnancy outcomes in patients with RRF is limited and has mainly focused on classifying patients for better clinical management, ignoring the effects of relevant immune factors on pregnancy outcomes of the patients. Bruno et al. (25) used machine learning to stratify patients with RPL into different risk categories, validated their appropriate prognosis and potential treatments through diagnostic workup, provided a decision-support system tool to stratify RPL patients, and objectively addressed their appropriate clinical management. Immune factors were not accounted for and pregnancy outcomes were not predicted. Li et al. (26) suggested that RPL may be related to abnormally elevated amounts of uterine natural killer (uNK) cells. They pointed out the difficulty of counting uNK and stromal cells under histochemical sections, because of the close morphological proximity of stromal cells to epithelial cells. This paper was the first to report on the ability to distinguish between different cell morphologies and accurately count them using image recognition techniques. Researchers can greatly benefit from this method in analyzing immunohistochemical images. Nevertheless, its application is limited and is unable to provide predictions, based on cell counts alone. Mora-Sanchez et al. (27) concluded that the degree of allelic sharing of human leukocyte antigen (HLA) genes is related to RPL, combining immunogenetics with A.I. to create a personalized tool to elucidate the genetic causes of unexplained infertility and a gamete matching platform that could improve pregnancy success.

The representative literature on the development of predictive models for pregnancy outcomes in recent infertile and patients with RRF is summarized in Table 1. Notably, a limited amount of models were observed that were established for the prediction of pregnancy outcome of patients with RRF. The published models were based only on genetic markers or common indices. To investigate the impact of immune factors on pregnancy outcomes in patients with RRF, we applied A.I. for the analysis of the medical information of patients with RRF, including immune indices for pregnancy outcomes prediction.

**Table 1:** Recent representative literature related to predictive models for patients with infertility or RRF.

**References**	**Sample size**	**Study design**	**List of selected features**	**Attribute/feature selection technique used**	**Validation (training/test procedure)**	**Modeling method**	**IVF/ICSI or RRF**	**Immune factors**	**Outcome**	**Performance reported**
Ramos-Medina et al. (28)	428	Retrospective cohort study	7 attributes	No feature selection	Did not mention	LR	RM, RIF	Yes	Clinical pregnancy, live birth	No report
Dhillon et al. (29)	12,638	Retrospective cohort study	8 attributes	No feature selection	9,915 of the data were used for training and 2,723 for testing	LR	IVF/ICSI	No	Live birth	AUC 0.62
Milewski et al. (30)	1,995	Retrospective cohort study	20 attributes	PCA, principal component analysis	Train-test	ANN	IVF	No	Clinical pregnancy	AUC 0.666
Vaegter et al. (31)	8,182	Prospective cohort study	36 attributes	Bivariate GEE regression	70% of the data were used for training and 30% for testing	GEE multivariate regression	IVF/ICSI	No	Live birth	Accuracy 0.67
Hafiz et al. (23)	486	Cross-sectional study	29 attributes	No feature selection	Five-fold cross validation	SVM, 1NN, RF, RPART, Adaboost	IVF/ICSI	No	Pregnancy	SVM: AUC 0.576, Accuracy 68.30%; 1NN: AUC 0.500, Accuracy 64.84% RF: AUC 0.842, Accuracy 83.96; RPART: AUC 0.821, Accuracy 83.56%; Adaboost: AUC 0.475, Accuracy 66.99%;
Hassan et al. (21)	1,048	Retrospective cohort study	25 attributes	Hill climbing wrapper algorithm	3/4 of the data were used for training and 1/4 for testing	MLP, SVM, C4.5, CART, RF	IVF	No	Pregnancy	MLP: AUC 0.991, Accuracy 97.77%; SVM: AUC 0.993, Accuracy 98.01%; C4.5: AUC 0.966, Accuracy 93.21%; CART: AUC 0.97, Accuracy 95.24%; RF: AUC 0.992, Accuracy 98.83
Ghaeini et al. (24)	251	Retrospective cohort study	9 attributes	No feature selection	70% of data were randomly selected for training, 15% for validation, and 15% for testing the model.	DT, SVM	ICSI	No	Clinical pregnancy	DT Accuracy 70.3%; SVM Accuracy 75.7%
Blank et al. (7)	1,052	Retrospective cohort study	32 attributes	No feature selection	Train-test	RF, LR	IVF/ICSI	No	Pregnancy	RF: AUC 0.74; LR: AUC 0.66
Vogiatzi et al. (8)	426	Retrospective cohort study	118 attributes	either *t*-test or **χ**^**2**^-test	70% of the data were used for training and 30% for the testing	ANN	IVF	No	Live birth	Accuracy 75.7%
Qiu et al. (32)	7,188	Retrospective cohort study	8 attributes	No feature selection	Five-fold cross-validation	LR, RF, SVM, XGBoost	IVF/ICSI	No	Live birth	LR AUC 0.72; RF AUC 0.73; SVM AUC 0.72; XGBoost AUC 0.74.
Itzhaki et al. (33)	72	Retrospective cohort study	13 attributes	RReliefF algorithm	Data were randomly split into a training set (70% of the data) and a test set (30%)	LR, SVM, NN	IVF	No	Positive beta-hCG, Clinical pregnancy, Live births	Positive beta-hCG: LR Accuracy 53%, SVM Accuracy 59%, NN Accuracy 85%; Clinical pregnancy, LR Accuracy 58%, SVM Accuracy 63%, NN Accuracy 90%; Live births, LR Accuracy 55%, SVM Accuracy 58%, NN Accuracy 87%.
Bruno et al. (25)	734	Retrospective cohort study	43 attributes	The most recent international guidelines of the ESHRE	k-fold cross-validation	SVM	RPL	No	Patients with RPL are classified into different risk levels	Accuracy 81.71%

*SVM, support vector machines; RPART, recursive partitioning; RF, random forest; Adaboost, adaptive boosting; 1NN, one-nearest neighbor; MLP, multilayer perceptron; C4.5; CART, classification and regression trees; LR, logistic regression; NN, neural network; ANN, artificial neural network; XGBoost, extreme gradient boosting; GEE, generalized estimating equations; RRF, recurrent reproductive failure; RPL, Recurrent pregnancy loss; RM, recurrent miscarriage; RIF, Recurrent implantation failure; ESHRE, European Society of Human Reproduction and Embryology*.

## Experimental Methods

### Data Acquisition

Medical data from 942 patients with RRF, including RPL or RIF, who visited the Fertility Center of Shenzhen Zhongshan Urology Hospital, China, from February 2015 to November 2019, were retrospectively extracted from the electronic medical record (EMR) system. RPL is defined as two or more spontaneous abortions prior to 20 weeks of gestation. RIF refers to infertile couples who experienced failure to conceive after multiple IVF cycles. During the process of diagnosis and laboratory detection, the medical data were generated and stored in the EMR system. Out of 942 samples, 381 were excluded, due to missing values. Finally, 561 data samples were included in the analysis.

### Data Processing

Data processing, model design, and programming were all carried out in MATLAB 2017b (The MathWorks, Inc.). Due to diversity in clinical testing and reporting period, concatenation of data within 90 days prior to serum human chorionic gonadotropin (hCG) test was performed for each patient. *Z*-score normalization was used for numerical data and one-hot encoding for categorical data.

### Model Training and Performance

The initialization of the dictionary matrix (Wd) was carried out using uniformly distributed numbers. Each column of Wd was normalized to a magnitude of 1. The processed and normalized data set (X) and dictionary matrix were used as input into the Iterative Shrinkage and Thresholding-based Algorithm with coordinate descent to obtain the sparse representation (Z) (34). Tanh and ReLu were selected as the activation functions of the hidden layers for the sparse representation. Softmax was used in the output layer for linear classification to obtain the prediction results. The cost of prediction was calculated using the sum of least squares between the prediction result and the true label. The Wd matrix was updated through backpropagation. Forward- and back-propagation were repeated until the optimal dictionary matrix was obtained (i.e., lowest cost). The data set was divided into training data set (90%) and testing data set (10%) for each data panel. The performance of the model was evaluated on the testing data set. The evaluation metrics included receiver operating characteristic (ROC) curves, accuracy, sensitivity, and specificity. The true positive (TP), true negative (TN), false positive (FP), and false negative (FN) rates were used for the calculation of the abovementioned metrics as follows:

$$\begin{array}{l} Accuracy=\;\frac{TP+TN}{TP+TN+FP+FN}\\ Sensitivity=\frac{TP}{TP+FN}\\ Specificity=\frac{TN}{TN+FP} \end{array}$$

## Results

### Clinical Characteristics of Samples

Following the literature review and in combination with the expertise of clinicians, six panels with 64 variables were considered as input variables. Three immune-related data panels, including the autoantibodies, peripheral immunology, and endometrial immunology panels were considered. Other IVF-related data panels contributing to the pregnancy outcome, including basic characteristics, hormones, and embryo panels were also considered. The clinical characteristics used in this study along with their respective description explaining their physical implications, type of the values, and their range in the collected data set are listed in Table 2. The mean age at the time of conception was 34.67 years and significantly different between the live birth group and non-live birth group. The average body mass index (BMI) was 21.69 kg/m^2^, with no statistically significant differences detected (21.55 vs. 21.79). Statistically significant differences were detected between some of the 64 variables, between the groups of patients who did and did not achieve a live birth (Table 2).

**Table 2:** Definition and data range of variables in the model.

**Field**	**Definition of field**	**Data range/class**	**Total *N* = 561 Mean ± SD/*N* (%)**	**Live birth *N* = 237 Mean ± SD/*N* (%)**	**No live birth *N* = 324 Mean ± SD/*N* (%)**	***P*-value**
**Basic characteristics panel**						
Female age (years)	Female age at time of conception	19–47	34.67 ± 4.39	33.73 ± 4.08	35.35 ± 4.48	<0.001
Female BMI (kg/m^2^)	Female body mass index	15.2–48.68	21.69 ± 3.01	21.55 ± 3.27	21.79 ± 2.80	0.166
Kayrotype of couple	Kayrotype analysis of couple	{Normal, abnormal}	Abnormal: 48 (8.60%) Normal: 510 (91.40%)	Abnormal: 22 (9.36%) Normal: 213 (90.64%)	Abnormal: 26 (8.05%) Normal: 297 (91.95%)	0.585
**Autoantibodies panel**						
aβ 2 GPI-IgM (U/ml)	Concentration of aβ 2 GPI-IgM	0.27–287	10.53 ± 19	12.9 ± 20.48	8.79 ± 17.68	0.418
aβ 2 GPI-IgG (U/ml)	Concentration of aβ 2 GPI-IgG	0.1–133.87	1.25 ± 5.86	1.3 ± 8.36	1.21 ± 2.92	<0.001
aCL-IgM (MPL)	Concentration of anti-cardiolipin antibody -IgM	0.13–104.75	4.69 ± 4.46	4.68 ± 4.62	4.7 ± 4.35	0.690
aCL-IgG (GPL)	Concentration of anti-cardiolipin antibody -IgG	0.23–102	5.16 ± 5.21	6.15 ± 3.84	4.43 ± 5.91	<0.001
aTG (IU/ml)	Concentration of anti-thymocyte globulin	0–1,801	87.25 ± 191.56	96.37 ± 204.4	80.57 ± 181.63	0.246
aTPO (IU/ml)	Concentration of anti-thyroidperoxidase antibodies	0–1,300	34.3 ± 75.4	35.44 ± 63.79	33.46 ± 82.95	0.393
SSA (U/ml)	Concentration of SSA	0–251	12.2 ± 18.66	13.36 ± 23.15	11.36 ± 14.50	0.471
SSB (U/ml)	Concentration of SSB	1–430	8.26 ± 8.53	8.45 ± 11.17	8.11 ± 5.9	0.060
Sm (U/ml)	Concentration of Sm	1–207	6.5 ± 4.8	6.99 ± 5.39	6.14 ± 4.3	0.169
RNP (U/ml)	Concentration of ribonucleoprotein	1–754	23.36 ± 27.12	25.23 ± 23.63	21.98 ± 29.37	0.008
Scl-70 (U/ml)	Concentration of Scl-70	1–212	15.2 ± 15.98	14.97 ± 13.06	15.37 ± 17.84	0.596
Jo1 (U/ml)	Concentration of Jo1	2–384	19.73 ± 28.13	19.96 ± 25.72	19.55 ± 29.81	0.862
dsDNA (U/ml)	Concentration of double-stranded DNA	0–112	16.09 ± 14.45	15.76 ± 15.61	16.33 ± 13.56	0.097
Centromeric B (U/ml)	Concentration of centromeric B	0–232	13.32 ± 14.7	14.03 ± 18.42	12.81 ± 11.24	0.940
histones (U/ml)	Concentration of histones	1–78	8.24 ± 5.8	8.07 ± 5.27	8.37 ± 6.16	0.774
**Peripheral immunology panel**						
D2 (ng/ml)	Concentration of D-dimer	45.36–30161.82	224.73 ± 188.95	228.1 ± 215.39	222.26 ± 167.29	0.809
ADP (%)	Platelet aggregation rate when ADP is used as an aggregator	5.7–98.9	75.82 ± 13.31	76.17 ± 12.81	75.56 ± 13.67	0.860
Col (%)	Platelet aggregation rate when Col is used as an aggregator	0.1–100	75.44 ± 20.15	75.46 ± 20.28	75.42 ± 20.08	0.406
ARA (%)	Platelet aggregation rate when ARA is used as an aggregator	0–100	59.05 ± 36.07	59.04 ± 34.19	59.06 ± 37.43	0.234
IgG T (%)	The percentage of IgG^+^ T cells in T cells	0.1–100	41.98 ± 35.35	47.13 ± 34.53	38.22 ± 35.52	0.001
IgG B (%)	The percentage of IgG^+^ B cells in B cells	0.8–100	62.16 ± 29.66	66.46 ± 27.24	59.01 ± 30.98	0.006
IFN-r (%)	The percentage of IFN-r^+^ Th cells in Th cells	3.6–67.8	22.53 ± 7.6	22.19 ± 6.82	22.78 ± 8.12	0.824
TNF-a (%)	The percentage of TNF-a^+^ Th cells in Th cells	5.1–84.4	38.88 ± 9.74	37.4 ± 8.88	39.97 ± 10.19	0.003
NK cytotoxicity 50:1	NK cytotoxicity to K562 at E: T ratio of 50:1	3.1–79.7	34.57 ± 12.5	36.39 ± 11.32	33.24 ± 13.15	0.005
NK cytotoxicity 25:1	NK cytotoxicity to K562 at E: T ratio of 25:1	1.4–76.2	23.42 ± 10.39	24.95 ± 9.98	22.3 ± 10.56	0.005
T (%)	The percentage of T cells in CD45^+^ lymphocytes	35.81–999.62	126.23 ± 194.65	138.04 ± 204.95	117.59 ± 186.61	<0.001
Tc (%)	The percentage of Tc cells in CD45^+^ lymphocytes	11.37–59.34	27.18 ± 6.1	27.08 ± 5.84	27.26 ± 6.3	0.908
Th (%)	The percentage of Th cells in CD45^+^ lymphocytes	16.77–62.96	37.04 ± 5.79	37.13 ± 5.88	36.98 ± 5.74	0.765
NK (%)	The percentage of NK cells in CD45^+^ lymphocytes	1.32–54.99	15.18 ± 5.07	15.02 ± 4.98	15.29 ± 5.14	0.674
B (%)	The percentage of B cells in CD45^+^ lymphocytes	3.68–33.49	13.72 ± 3.77	13.76 ± 3.7	13.68 ± 3.83	0.868
CD4/CD8	The ratio of Th cells and Tc cells	0.34–3.62	1.47 ± 0.49	1.48 ± 0.48	1.46 ± 0.5	0.346
T (No.)	The absolute number of T cells per 100 μl blood	362.78–5999.08	1582.8 ± 487.26	1557.77 ± 475.69	1601.1 ± 495.48	0.213
Tc (No.)	The absolute number of Tc cells per 100 μl blood	104.86–3142.96	614.74 ± 231.03	600.82 ± 199.57	624.92 ± 251.38	0.872
Th (No.)	The absolute number of Th cells per 100 μl blood	241.58–3175.38	844.39 ± 302.62	836.67 ± 327.13	850.03 ± 283.73	0.277
NK (No.)	The absolute number of NK cells per 100 μl blood	33.95–1907.72	344.6 ± 160.4	335.25 ± 155.15	351.45 ± 164.04	0.133
B (No.)	The absolute number of B cells per 100 μl blood	61.78–1731.29	320.43 ± 167.27	318.54 ± 180.53	321.82 ± 157.14	0.639
**Endometrial immunology panel**						
HE	Histological dating	{Inconformity, early, mid, late}	Inconformity: 27 (20.00%) Early: 1 (0.74%) Mid: 106 (78.52%) Late: 1 (0.74%)	Inconformity: 2 (25.00%) Early: 0 (0%) Mid: 6 (75.00%) Late: 0 (0%)	Inconformity: 25 (19.69%) Early: 1 (0.79%) Mid: 100 (78.74%) Late: 1 (0.79%)	0.949
CD56 (%)	The percentage of CD56^+^ cells in total endometrial cells	0.5–58.77	13.13 ± 6.94	13.8 ± 7.35	12.64 ± 6.6	0.051
Foxp3 (%)	The percentage of Foxp3^+^ cells in total endometrial cells	0.01–1.11	0.1 ± 0.06	0.1 ± 0.06	0.1 ± 0.06	0.891
CD68 (%)	The percentage of CD68^+^ cells in total endometrial cells	0.15–12.32	2.22 ± 0.95	1.95 ± 0.94	2.41 ± 0.92	<0.001
CD163 (%)	The percentage of CD163^+^ cells in total endometrial cells	0.5–10	2.64 ± 1.2	2.81 ± 1.34	2.53 ± 1.08	0.015
CD1a (%)	The percentage of CD1a^+^ cells in total endometrial cells	0–0.612	0.07 ± 0.06	0.07 ± 0.05	0.08 ± 0.06	0.030
CD83 (%)	The percentage of CD83^+^ cells in total endometrial cells	0.09–11.37	2 ± 1.01	1.88 ± 1.1	2.08 ± 0.93	<0.001
CD57 (%)	The percentage of CD57^+^ cells in total endometrial cells	0.02–2.66	0.39 ± 0.24	0.35 ± 0.22	0.41 ± 0.25	0.002
CD8 (%)	The percentage of CD8^+^ cells in total endometrial cells	0.53–18.27	3.14 ± 1.65	2.8 ± 1.6	3.38 ± 1.65	<0.001
CD138	The intensity of CD138^+^ cells in endometrial tissue	{–, ±, +}	–: 496 (96.88%) ±: 2 (0.39%) +: 14 (2.73%)	–: 190 (98.45%) ±: 2 (1.04%) +: 1 (0.52%)	–: 306 (95.92%) ±: 0 (0%) +: 13 (4.08%)	0.004
**Hormone panel**						
FSH (mIU/ml)	Concentrationn of follicle-stimulating hormone	0.97–59.62	7.31 ± 3.49	7.3 ± 4.24	7.31 ± 2.82	0.593
LH (mIU/ml)	Concentrationn of luteal hormone	0.35–48.96	5.14 ± 3.34	5.51 ± 4.26	4.87 ± 2.42	0.069
E2 (pg/ml)	Concentration of estrogen	0.29–1,778	52.12 ± 69.45	46.02 ± 36.55	56.57 ± 85.67	0.773
P (ng/ml)	Concentration of progesterone	0.03–59.02	0.83 ± 2.59	0.81 ± 2.49	0.84 ± 2.66	0.004
PRL (ng/ml)	Concentration of prolactin	0.3–1,249	37.69 ± 90.22	36.14 ± 68.48	38.83 ± 103.35	0.023
T (ng/ml)	Concentration of testerone	0–71.42	1.63 ± 6.07	1.47 ± 5.59	1.75 ± 6.4	0.083
TSH (μIU/ml)	Concentration of thyroid stimulating hormone	0.01–25.33	2.32 ± 1.01	2.29 ± 1.04	2.35 ± 1	0.138
FT3 (pg/ml)	Concentration of free triiodothyronine	1.01–301.2	3.11 ± 0.5	3.08 ± 0.46	3.14 ± 0.52	0.370
FT4 (ng/dl)	Concentration of free thyroxine	0.71–84.88	2.01 ± 2.42	1.88 ± 2.37	2.1 ± 2.45	0.002
**Embryo panel**						
ways to conceive	IVF-ET or natural conception	{IVF-ET, natural conception}	IVF-ET: 517 (92.16%) Natural conception: 44 (7.84%)	IVF-ET: 215 (90.72%) Natural conception: 22 (9.28%)	IVF-ET: 302 (93.21%) Natural conception: 22 (6.79%)	0.278
Endometrial preparation programs	Endometrial preparation programs during IVF-ET cycle	{Hormone-replacement cycle, natural cycle, others}	Hormone-replacement cycle: 200 (43.67%) Natural cycle: 113 (24.67%) Others: 145 (31.66%)	Hormone-replacement cycle: 79 (47.59%) Natural cycle: 46 (27.71%) Others: 41 (24.70%)	Hormone-replacement cycle: 121 (41.44%) Natural cycle: 67 (22.95%) Others: 104 (35.62%)	0.050
Fertilization way	Fertilization method to get embryo	{ICSI, IVF}	ICSI: 154 (32.56%) IVF: 319 (67.44%)	ICSI: 54 (27.69%) IVF: 141 (72.31%)	ICSI: 100 (35.97%) IVF: 178 (64.03%)	0.059
Type of embryo	Type of embryo	{Blastosphere, cleavage stage embryo}	Blastosphere: 324 (62.43%) Cleavage stage embryo: 195 (37.57%)	Blastosphere: 148 (69.48%) Cleavage stage embryo: 65 (30.52%)	Blastosphere: 176 (57.52%) Cleavage stage embryo: 130 (42.48%)	0.006
Type of transfer	Embryo transfer or frozen embryo transfer	{ET, FET}	ET: 53 (10.27%) FET: 463 (89.73%)	ET: 40 (18.96%) FET: 171 (81.04%)	ET: 13 (4.26%) FET: 292 (95.74%)	<0.001
No. of transferred embryo	The number of transferred embryos in one transfer cycle	1–3	1.8 ± 0.55	1.88 ± 0.57	1.73 ± 0.53	0.012
Quality of embryo	Quality of transferred embryo	{Sequence 1,2,3,4,5}^a^	1: 624 (68.95%) 2: 244 (26.96%) 3: 32 (3.54%) 4: 4 (0.44%) 5: 1 (0.11%)	1: 296 (75.32%) 2: 84 (21.37%) 3: 12 (3.05%) 4: 1 (0.25%) 5: 0 (0%)	1: 328 (64.06%) 2: 160 (31.25%) 3: 20 (3.91%) 4: 3 (0.59%) 5: 1 (0.2%)	0.006

### Model Performance on Immunological Data Panels

**Figure 1:**
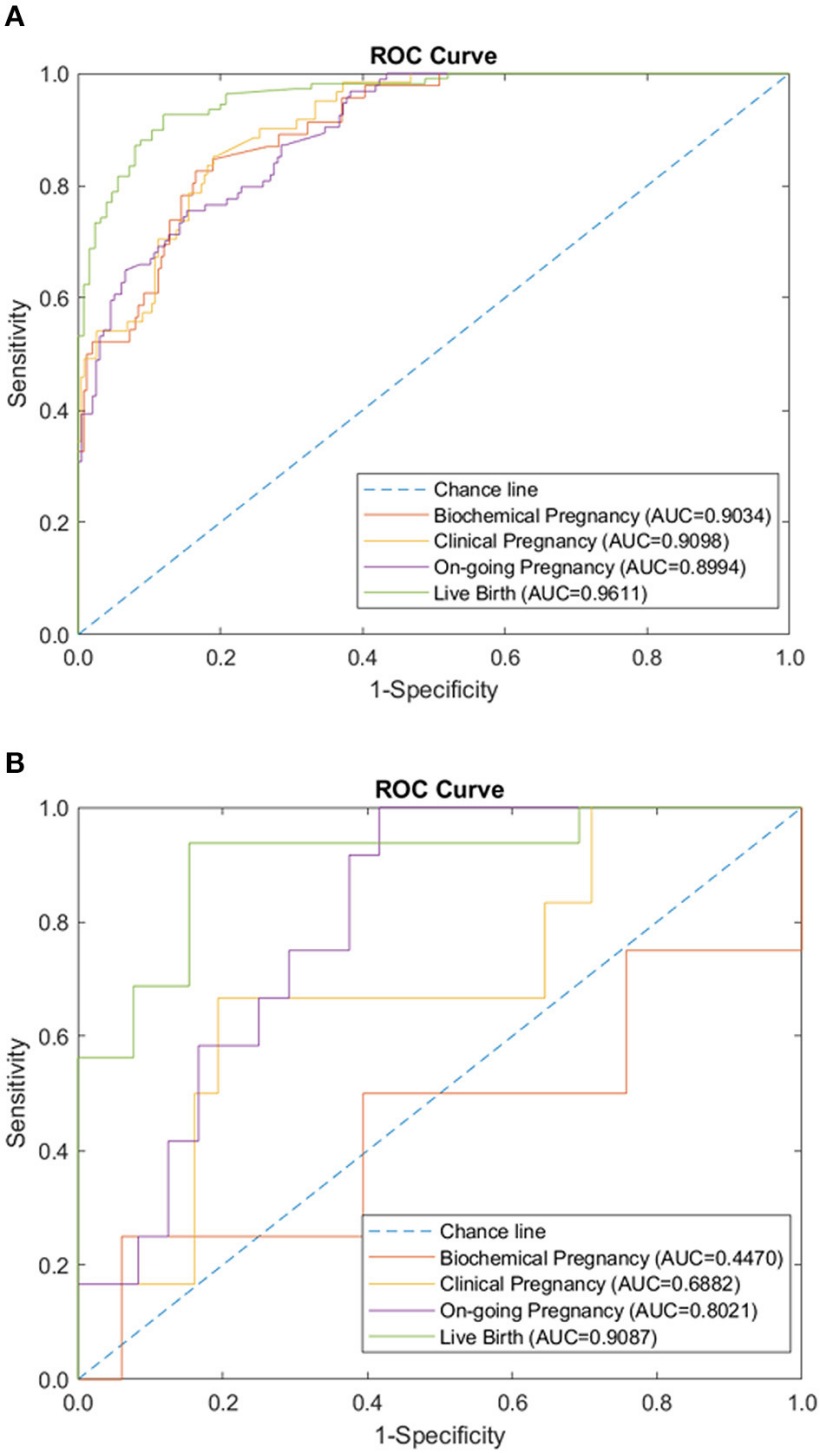
Autoantibodies panel performance of sparse coding in predicting pregnancy outcomes at different pregnancy periods. **(A)** ROC plot of the training data set. **(B)** ROC plot of the testing data set.

**Figure 2:**
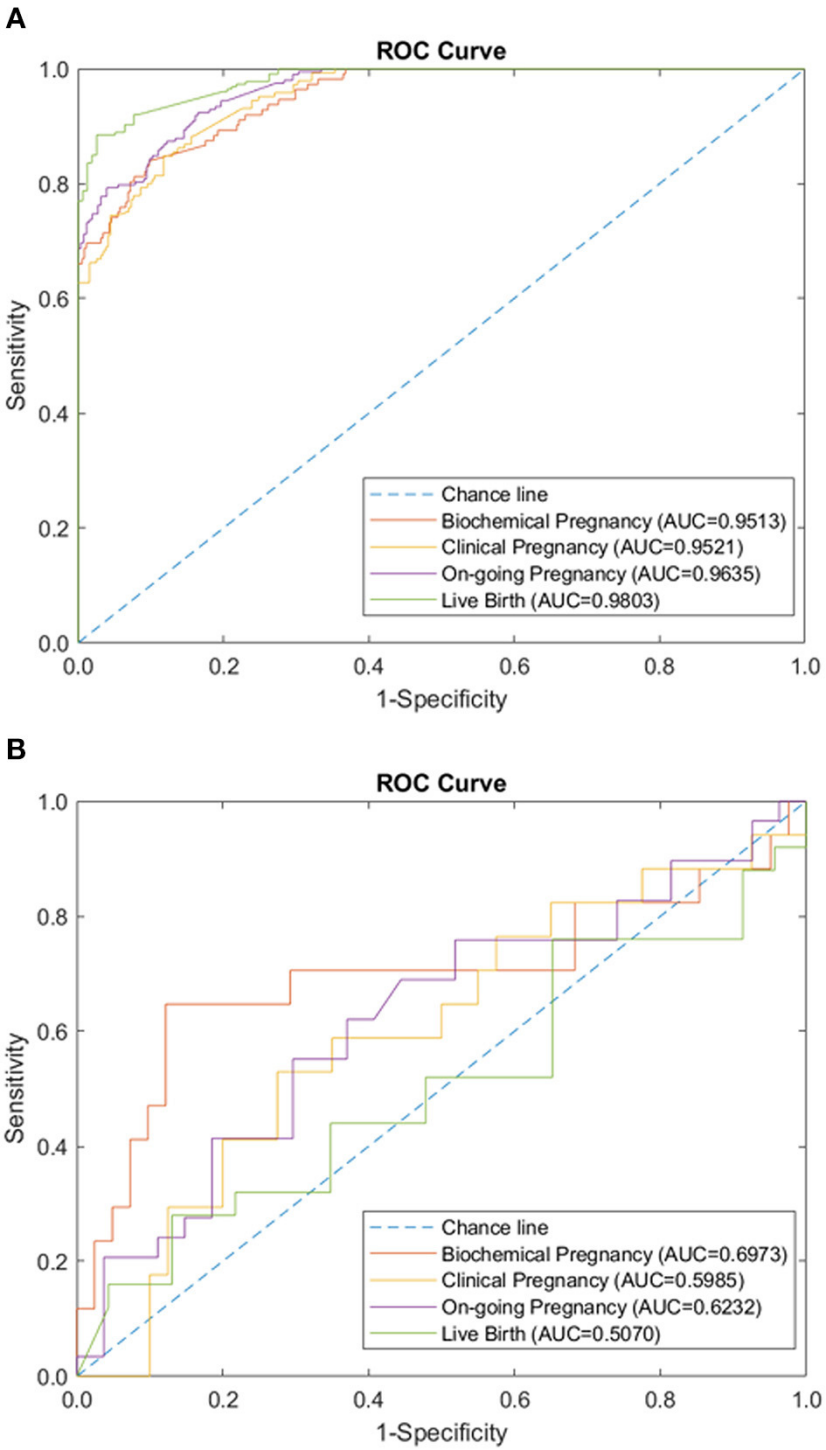
Peripheral immunology panel performance of sparse coding in predicting pregnancy outcomes at different pregnancy periods. **(A)** ROC plot of the training data set. **(B)** ROC plot of the testing data set.

**Figure 3:**
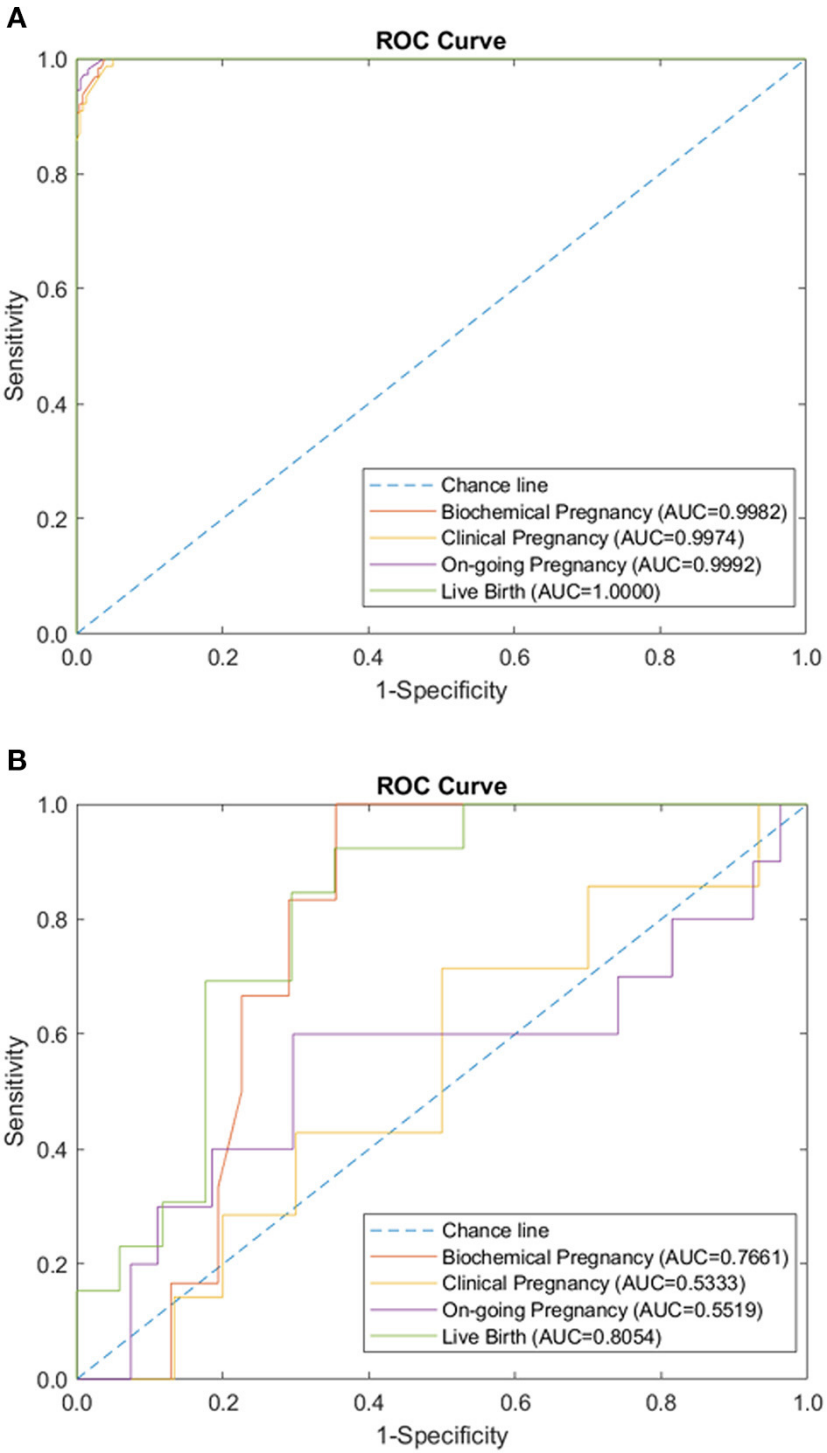
Endometrial immunology panel performance of sparse coding in predicting pregnancy outcomes at different pregnancy periods. **(A)** ROC plot of the training data set. **(B)** ROC plot of the testing data set.

**Figure 4:**
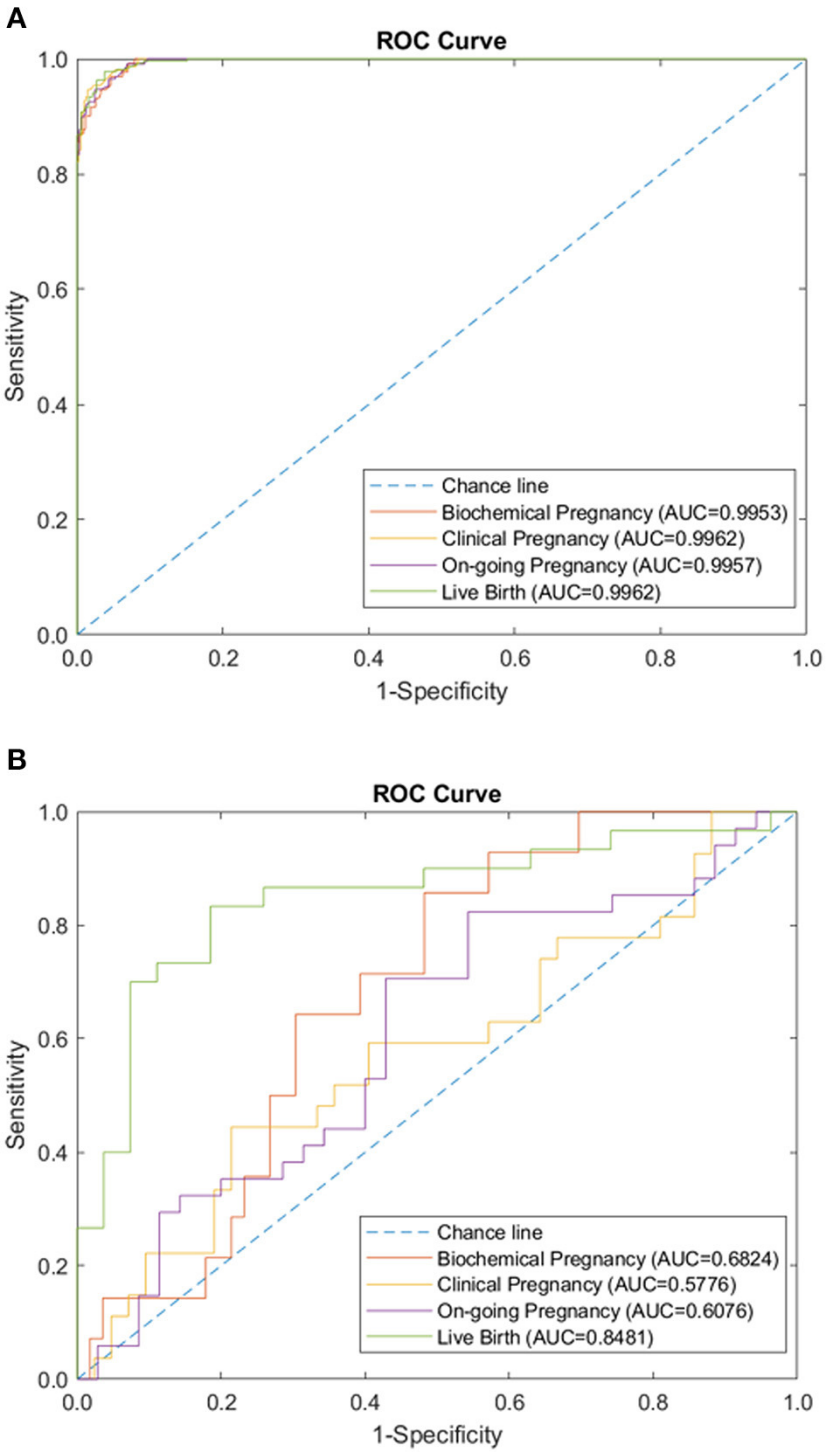
Combination of immunology-related panels (autoantibodies, peripheral immunology, and endometrial immunology) performance of sparse coding in predicting pregnancy outcomes at different pregnancy periods. **(A)** ROC plot of the training data set. **(B)** ROC plot of the testing data set.

We tested the sparse coding model using various data panels including autoantibodies (Figure 1), peripheral immunology (Figure 2), endometrial immunology (Figure 3), and the combination of all three immunological data panels (Figure 4). A summary of prediction accuracy using various data panels is shown in Table 3. Four output labels were used for prediction, namely, biochemical pregnancy, clinical pregnancy, ongoing pregnancy, and live birth. The ROC curves during the model training generally had AUC values exceeding 0.9, for each immunological panel. The AUC values decreased during testing. In terms of predicting biochemical pregnancy, the AUC of prediction using the endometrial immunology panel was higher (AUC = 0.766, accuracy: 73.0%) compared to the AUC of the panel using autoantibodies (AUC = 0.447, accuracy: 70.3%) and peripheral immunology panel (AUC = 0.697, accuracy: 72.4%). The AUC of prediction using the autoantibodies panel was higher in predicting clinical pregnancy (AUC = 0.688, accuracy: 78.4%), ongoing pregnancy (AUC = 0.802, accuracy: 75.0%), and live birth (AUC = 0.909, accuracy: 89.7%), compared to the AUC of the panels using peripheral immunology and endometrial immunology panel. Combining all three immunological data panels did not result in an increase of the AUCs of all four pregnancy outcomes.

**Table 3:** Summary of training and testing accuracy of models on various data panels.

**Data panels**	**Pregnancy outcome**	**Training accuracy (%)**	**Testing accuracy (%)**
Autoantibodies	Biochemical pregnancy	86.7	70.3
	Clinical pregnancy	84.9	78.4
	Ongoing pregnancy	83.1	75.0
	Live birth	89.0	89.7
Peripheral immunology	Biochemical pregnancy	93.0	72.4
	Clinical pregnancy	87.4	68.4
	Ongoing pregnancy	87.4	55.4
	Live birth	92.2	54.2
Endometrial	Biochemical pregnancy	98.0	73.0
immunology	Clinical pregnancy	97.0	59.5
	Ongoing pregnancy	98.3	62.2
	Live birth	100	76.7
Combined	Biochemical pregnancy	96.1	65.7
immunology-related	Clinical pregnancy	97.1	62.3
panels	Ongoing pregnancy	95.6	55.1
	Live birth	97.0	79.0
Combined	Biochemical pregnancy	100.0	68.1
immunology-related	Clinical pregnancy	100.0	70.6
panels and IVF-related	Ongoing pregnancy	100.0	68.7
panels	Live birth	100.0	71.4

### Performance of Model on Combined Data Panels

**Figure 5:**
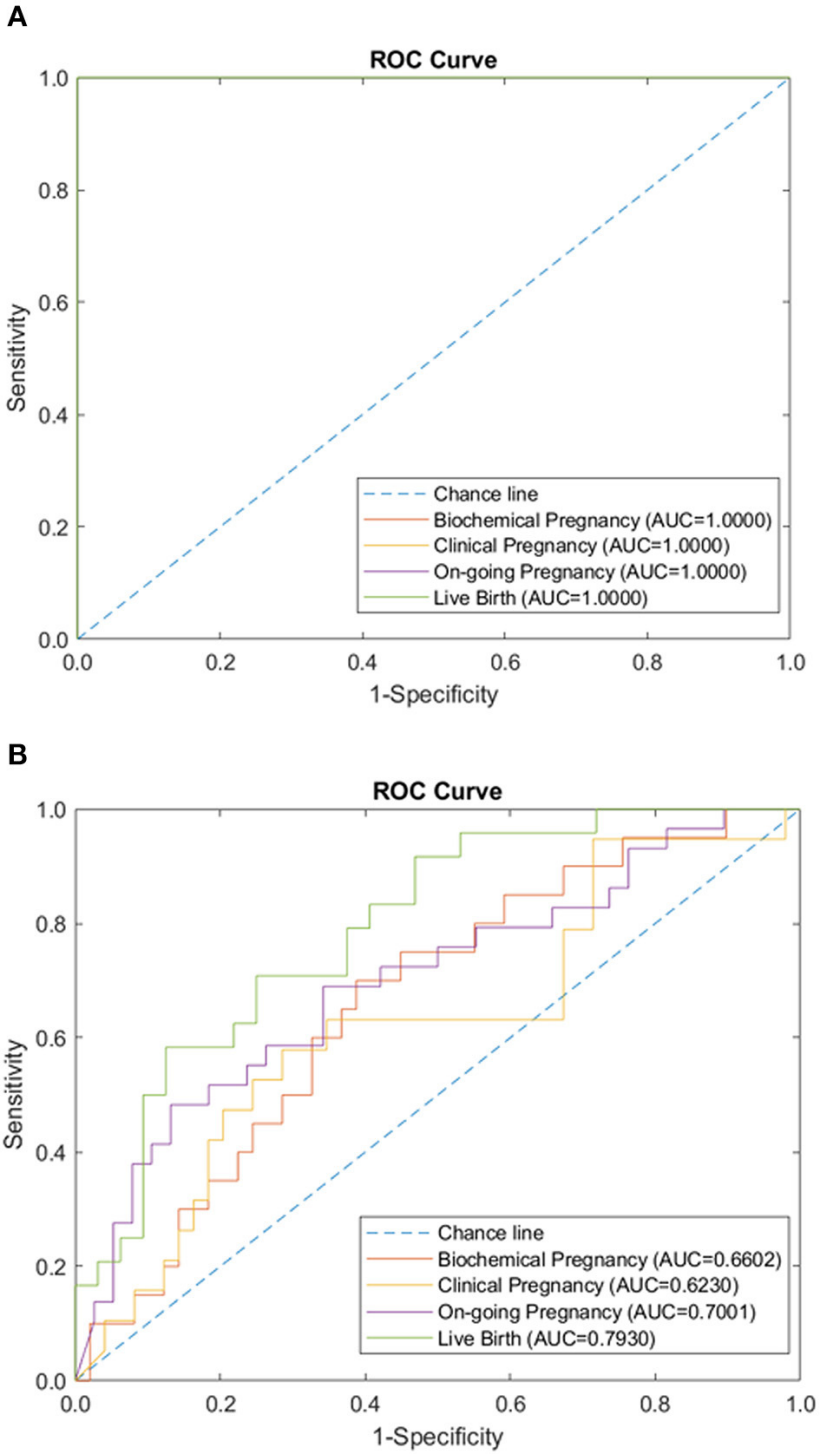
Combination of immunology and IVF-related panels (autoantibodies, peripheral immunology, endometrial immunology, basic patient characteristic, hormone level, and embryo parameter) performance of sparse coding in predicting pregnancy outcomes at different pregnancy periods. **(A)** ROC plot of the training data set. **(B)** ROC plot of the testing data set.

Additionally, the sparse coding model was tested using both IVF-related data panels and immunological data panels. The AUC during the training phase was 1.0 for the prediction of all four pregnancy outcomes, using combined data panels. The AUC during the testing phase ranged from 0.661 to 0.793, following a similar lower trend, as in the case of immunological data panels (Figure 5). The use of combined data panels did not significantly enhance the effect on the prediction of all four pregnancy outcomes.

## Discussion

A machine learning model was developed in this study for the prediction of the pregnancy outcomes for the patients with RRF at any gestational period, namely, biochemical pregnancy, clinical pregnancy, ongoing pregnancy, and live birth. The accuracy of the models for each stage in the testing data set ranged from 54.2 to 89.7%.

We observed that the performance of the endometrial immunology panel in biochemical pregnancy prediction was superior to the autoantibodies and peripheral immunology panel. Consistent with this result, it has been reported that implantation failure in ART is thought to be mainly due to impaired endometrial receptivity (35). In addition, implantation failure and miscarriage occurrence have been reported to have different mechanisms (36). Antiphospholipid syndrome (APS), which is characterized by the presence of anti-cardiolipin autoantibodies (ACAs), is the most common autoimmune disease associated with RPL (37). However, the association between ACAs and RIF is somewhat controversial (38). Anti-thyroid autoantibodies (ATA) have been also demonstrated to correlate with RPL, while, the association between ATA and RIF remains unclear (39–41). Our results showed that the AUC of the model using the autoantibodies panel in predicting clinical pregnancy, ongoing pregnancy, and live birth was the highest, while, the AUC for biochemical pregnancy prediction was the lowest. Subsequently, we can safely conclude that different models are appropriate for pregnancy outcome prediction at different pregnancy periods.

Machine learning algorithms have been widely used in many complex scenarios, such as image analysis, diagnosis, classification, and prognosis (42). Multiple machine learning techniques have been applied to improve the success rate of ART. The A.I. application in reproductive medicine has focused mainly on oocytes evaluation and selection (43), sperm analysis and selection (44), and embryo selection (45). A few studies have attempted to establish models for IVF outcome prediction (23, 46). Typical machine learning techniques such as Deep Artificial Neural Network (DANN) and Convolutional Neural Network (CNN) can be used to handle the high dimensionality data features, but very often these models are hard to interpret due to the “black-box” situation (47), which is usually not favored in biomedical applications. We adopted sparse coding which helps in the creation of an overcomplete information space composed of atom features with high dimensionality, which are critical to our model classifications. Simultaneously, the sparse representation of atoms can highlight the important features of patients using only a few atoms. It also enables us to visualize the features and interpret the classification or prediction results. To our knowledge, this is the first sparse coding-based prediction model based on reproductive big data including basic patient characteristics, hormone levels, immune status, and embryo parameter information for patients with RRF. This model represents an attempt at combining the reproductive immunology parameters with a machine learning algorithm.

In conventional clinical practice, clinicians can only provide the successful pregnancy probability to the patient according to the mean success rate of the fertility center. In addition to predicting pregnancy outcomes, clinicians are also concerned about developing effective treatment strategies based on the medical data of the patient. The models in the majority of the previous studies provided the live birth probability to the clinicians. Given the variation in the probability of success, the clinicians were unable to know how close the status of the patient is related to a successful pregnancy. The clinicians usually plan the treatment strategy according to the medical data of the patient and their experience on the underlying connection between different parameters. Interpreting the underlying relationships between medical data may influence the decision of the clinicians concerning treatment strategies. Future studies can include a thorough analysis of the immune status of the patients, by comparing the atoms which contribute to a successful pregnancy, generated in the sparse representation and assist clinicians to develop more personalized treatment strategies based on the comparison result.

Several limitations of this study need to be considered. First, the entire data set of this study was derived from a single reproductive immunology center. Second, other factors that potentially affect pregnancy outcomes, such as lifestyle (e.g., smoking history) and family genetic history, were not taken into consideration in our study. Finally, the performance of our model is related to the quantity and quality of the data. Therefore, the model presented here needs further study with more multi-center clinical data before full implementation in a clinical setting. Moreover, the current A.I. is mainly used as a support system to improve the accuracy and efficacy of the clinicians, rather than a stand-alone decision-making system. The clinicians should collaborate with algorithm engineers to continually optimize the model, as it is applied in clinical work.

## Data Availability Statement

The raw data supporting the conclusions of this article will be made available by the authors, without undue reservation.

## Ethics Statement

The studies involving human participants were reviewed and approved by Ethics Committee of Shenzhen Zhongshan Urology Hospital, Shenzhen, China. Written informed consent for participation was not required for this study in accordance with the national legislation and the institutional requirements.

## Author Contributions

WT, YZe, and WL were involved in the study design. CH, ZX, and WL were involved in the organization of the entire project, data analysis with a clinical perspective, and manuscript writing. DT, CY, and LW were involved in the establishment of the algorithm. YZh, YL, SY, and LD were involved in collecting and preparing the clinical data. ZL was mainly involved in the literature review about A.I. and reproductive medicine. All authors contributed to the article and approved the submitted version.

## Conflict of Interest

DT, CY, LW, and WL were employed by the company ALOM Intelligence Limited, Hong Kong, China. The remaining authors declare that the research was conducted in the absence of any commercial or financial relationships that could be construed as a potential conflict of interest.
